# Thrombotic Processes in Multiple Sclerosis as Manifestation of Innate Immune Activation

**DOI:** 10.3389/fneur.2014.00119

**Published:** 2014-07-07

**Authors:** Tatiana Koudriavtseva

**Affiliations:** ^1^Unit of Neurology, Multiple Sclerosis Center, Regina Elena National Cancer Institute, Rome, Italy

**Keywords:** multiple sclerosis, thrombosis, innate immunity, autoimmunity, infection

## Introduction

Multiple sclerosis (MS) is a chronic demyelinating inflammatory disease of the central nervous system (CNS) affecting prevalently young adults (1, 2). The pathogenesis of MS has long been attributed to self-reactive T cells but recently the relevant role of B cells has also been recognized (2, 3). Furthermore, it has been demonstrated that innate immunity has a pivotal role in the beginning and in advanced stages of MS (4–6).

Innate immunity represents the immediate non-specific defense against infections and dangerous agents acting through its essential arms such as inflammation and blood coagulation (7–10). The coagulant processes are normally balanced by the natural anti-coagulant system needed to limit the host damage, and their imbalance leads to venous thrombosis (7, 8).

Many evidences reported below support a significant presence of local and systemic thrombotic events in MS confirming the global over-stimulation of innate immunity for both its inflammatory and coagulant components.

## Background

### Venous thromboembolism and cerebral thrombosis in MS

Several extensive studies have shown the association between venous thromboembolism and MS from its early stages. In a large population-based cohort study evaluating data from a 30-year Danish National Registry, MS was associated with increased long-term risk of venous thromboembolism (11). Another paper, based on the review of one cohort study and three cross-sectional studies, found that early MS was associated with an increased risk of pulmonary embolism, deep venous thromboembolism, and stroke, both the latter persisting over time (12). Other large studies have shown an increased risk of venous thromboembolism in MS as well as in several other immune-mediated illnesses (13–15).

Cases of cerebral thrombosis have been also reported in MS, mainly attributed to the effect of lumbar puncture and methylprednisolone therapy, however, in some cases no pre-disposing condition was found (16–19).

Moreover, a collaborative genome-wide association study screen in MS patients, not only implicated a multitude of genes coding for cytokine pathway, co-stimulatory, and signal transduction molecules of immunological relevance, but also for a vascular cell adhesion molecule (VCAM1) mediating leukocyte–endothelial cell adhesion and signal transduction (20). Interestingly, thrombin is able to induce expression of functional forms of VCAM1 on endothelial cells (21) and this expression can be inhibited by the specific thrombin inhibitor, hirudin. In my opinion, these data may further support the association between coagulation system and inflammation as well as between components of innate and adaptive immunity in MS.

### Pro-thrombotic conditions and vascular dysfunctions in MS

There are several studies confirming platelet (22–24) and complement (25–27) involvement in MS, both essential for the innate immune response by linking inflammation and coagulation. Moreover, many vascular dysfunctions have been reported in MS. They have been largely resumed in the review of D’Haeseleer and colleagues (28) facilitating their discussion in the restricted space of this work.

#### Vascular risk factors in MS

Interestingly, in the review of D’Haeseleer and colleagues, all the vascular risk factors reported to be associated with MS such as reduced physical activity, smoking, endothelial dysfunction, platelet activation, thrombophilia, and hyper-homocysteine are essentially pro-thrombotic conditions. Conversely, other vascular risk factors such as atrial fibrillation, flutter, arterial hypertension, diabetes mellitus, dyslipidemia, and obesity have not been associated with MS (28).

#### Cerebral hypoperfusion in MS

Decreased cerebral blood volume and flow, as well as its prolonged brain mean transit time, were demonstrated in all forms of MS and even in clinically isolated syndrome, both in white and gray matter, by SPECT, PET, and dynamic susceptibility contrast-enhanced MRI (28). In my opinion, the widespread cerebral hypoperfusion in MS may be prevalently determined by the blood flow deceleration in the venous bed due to the inflammatory-thrombotic processes. This consequently leads to decreased arterial flow with reduced tissue oxygenation and energy metabolism clarifying the similarities between ischemic and MS lesions for both the histopathological features and enhanced expression of hypoxia-inducible factors mentioned by D’Haeseleer et al. This explanation of cerebral hypoperfusion appears to be more effective compared to both reduced axonal activity and decreased astrocyte energy metabolism proposed in the review as the possible explanation for hypoperfusion.

#### Venous blood drainage

According to D’Haeseleer et al., I think that MS could unlikely be caused by the obstructed extracranial venous drainage. The CCSVI theory explains the perivenular pattern of MS lesions by extravasation and degradation of erythrocytes due to blocked venous drainage with consequent iron deposition causing the inflammatory demyelinating lesions. However, the perivenular distribution of MS lesions, named “Dawson’s fingers,” prevalently around the ventricle-based brain veins, has long been thought to be the result of inflammation and coagulation around the major axis of medular veins (29).

Following the first single center study (30), many groups have tried to replicate their findings, but largely without success. Two recent studies from Italy (31) and Canada (32) provide further evidence to refute the idea of CCSVI as a cause of MS.

Even the short-lasting improvements reported by many operated patients that were considered placebo effect by the authors of the review (28), may be partly attributable to the intra-operatory intravenous and 3-week subcutaneous low-weight heparin therapy according to Zamboni’ protocol (33), since heparin has been used with some success in MS in the 60s (34, 35). However, further studies are needed in order to confirm this hypothesis.

Nevertheless, in my opinion, several studies carried out to test this theory have indirectly demonstrated the presence of the widespread endothelial damage likely due to the persistence of inflammatory-thrombotic processes. The emerging evidences on the link of jugular venous reflux with several CNS diseases have not clarified their cause–effect relationship (36), however, the venous dysfunctions seem to be the common consequence of inflammatory processes on the vascular endothelium rather than the unique anatomic cause of different disorders. Interestingly, a reduction in venous vasculature visibility on susceptibility-weighted imaging venography was already demonstrated, thus suggesting a correlation with both a decrease in metabolism and venous morphological alterations in the brain (37). These manifestations could correlate to endothelial injury determined by long-term inflammatory-thrombotic processes rather than to the stenosis of extracranial veins. The latter should determine brain venous engorgement in contrast with reduced visibility of venous vasculature.

More convincingly, Kirk and colleagues much earlier found the microvascular endothelial tight junctions abnormalities in vessels of any size, especially in active lesions as compared to normal appearing white matter, supporting a causative role of diffusible inflammatory mediators (38). In fact, the inflammatory factors increase the endothelial permeability and the expression of adhesion molecules determining the extravasation of immune cells and erythrocytes (7, 8).

### Role of coagulation system in MS and in experimental allergic encephalomyelitis

Many evidences in literature support the role of the coagulation system as an expression of innate immune activation both in MS and in experimental allergic encephalomyelitis (EAE).

In 1935, Putnam already proposed the pivotal role of venule thrombosis in MS (39). Another pathological study in acute MS reported fibrin deposition on endothelial cells in many thin veins and capillaries in areas without myelin damage or reactive parenchymal changes, with some thrombosed vessels or even intra-cerebral hemorrhage (40). Moreover, coagulation proteins were detected within chronic active plaques in MS (41).

Chapman highlighted the relevance of thrombin in inflammatory brain diseases (42, 43). Thrombin has numerous hormone-like functions modulating, among other, microglia and astrocytes (44). The activity of thrombin in the brain is regulated by thrombin inhibitors such as serum antithrombin III and brain protease nexin-1 secreted by glial cells and neurons that confirms the relevance of thrombin regulation in the brain (42, 43). An increase of brain protease nexin-1 levels was shown at early pre-clinical stages in EAE (45) while the plasma thrombin–antithrombin complexes increased immediately prior to EAE symptoms and decreased in relation to their improvement (46).

The activated microglia, a cellular component of innate immunity in the brain, along with fibrin deposition, were both found in acute/early MS and in rat EAE lesions, representing a first stage of tissue injury before active demyelination and massive T-cell infiltration (47). Fibrin deposition preceded and regulated inflammatory demyelination, as well as genetic or pharmacological fibrin depletion ameliorated both clinical symptoms and inflammatory response in EAE (48). Likewise, hirudin or recombinant activated protein C improved EAE and suppressed pro-inflammatory T-helper1 and T-helper17 cytokines in astrocytes and immune cells (41). The suppression of EAE by dermatan sulfate (49) or low doses of heparins (50) has been long demonstrated.

All reported here data highlight that the activation of coagulation system, linked to innate immunity, is a mandatory process during the early immune reaction toward the tissue damage of any kind. This reaction is independent of the underlying cause of injury and precedes a specific adaptive immune response in both MS and EAE.

## Discussion

Although the histopathological studies have only rarely found cerebral venous thrombosis in MS, there is convincing evidence supporting the presence of inflammatory-thrombotic processes during the overall course of the disease, from its early phases. This might confirm the relevant role of innate immunity in MS not only for its inflammatory component (4–6, 47), but also for the coagulant one. These manifestations are present both in the brain and at peripheral level reflecting a systemic stimulation of innate immunity by unknown exogenous factors that may be represented by viral infections (51). In fact, even if the etiology of MS still remains unexplained, viral infections are considered the main environmental factors related to MS as suggested by their association with disease exacerbations (52) as well as by many epidemiological studies and the common presence of oligoclonal IgG in the cerebrospinal fluid of MS patients (51) and by their relevance as non-heritable factors in gene–environment interactions (53).

The early systemic activation of innate immunity suggests a direct triggering by pathogens rather than by an indirect autoimmune CNS reaction. The innate immunity not only stimulates and modulates adaptive immunity at the beginning of disease but also mediates the neurodegenerative changes in progressive phase of MS (5, 6). While many efforts are aimed to better define the function of the innate immune cells in order to modulate their potential therapeutic action in MS (5, 6), the coagulant component of innate immunity, well studied in animals, is not sufficiently evaluated in humans.

The physiological innate immune response to infections does not usually determine the thrombotic manifestations due to an effective action of the natural anti-coagulant system. However, thrombotic manifestations can take place when the natural anti-coagulant system fails to counterbalance the prolonged or intense coagulation processes. These conditions may occur occasionally in immune-competent people during particularly severe infections, or may manifest persistently during chronic latent infections occurring in individuals with unregulated immunity and genetic or environmentally induced impairment of anti-coagulant system. MS and other immune-mediated disorders associated with thromboembolism may fall into this last category. However, there is no doubt that if the activation of innate immunity is directly triggered by viral infection, playing a protective role against infection, our therapeutic intervention should be not greater than necessary to compensate the reduced anti-coagulant capacity.

### Conclusion

Several evidences support persistent inflammatory-thrombotic processes in MS confirming the prolonged activation of innate immunity, likely by chronic latent infections. Experimental evidences coming from the literature here reported may open new therapeutic perspectives for MS patients. In fact, it is already possible to interfere with the coagulation system at various levels of the cascade, and clinical trials trying to transfer the promising results on EAE to humans are needed.

## Conflict of Interest Statement

No conflict of interest or financial interest is reported. T. Koudriavtseva reports consulting fees from Bayer Schering, and Institutional grant from Merck Serono, Biogen Idec, Novartis, Bayer Schering outside the submitted work.
